# One Health preparedness for Ebola disease caused by Bundibugyo virus: lessons from the 2026 outbreak centred in the Democratic Republic of the Congo

**DOI:** 10.1016/j.soh.2026.100170

**Published:** 2026-09-29

**Authors:** Yuxuan Shao, Jue Liu, Lei Zhou

**Affiliations:** a School of Public Health, Nanjing Medical University, Nanjing, China; b School of Public Health, Peking University, Beijing, China; c Chinese Center for Disease Control and Prevention & Chinese Academy of Preventive Medicine, Beijing, China

**Keywords:** Bundibugyo virus disease, One Health, Outbreak preparedness, Diagnostic readiness, Medical countermeasures, Infection prevention and control, Cross-border coordination

## Abstract

The 2026 outbreak of Bundibugyo virus disease (BVD), caused by Bundibugyo virus (BDBV), involved limited international exportation but remained centred in eastern Democratic Republic of the Congo (DRC). The outbreak tested whether earlier institutional mobilisation translated into timely field delivery. We conducted a structured narrative review of peer-reviewed research, official reports and operational guidance on epidemiology, diagnostics, medical countermeasures (MCMs), One Health surveillance, clinical care, infection prevention and control (IPC), community engagement and cross-border preparedness. Key institutional mechanisms for emergency decision-making, coordination and MCM prioritisation were activated earlier than during the 2013–2016 Ebola virus disease epidemic in West Africa, widening the window for action. Field delivery nevertheless remained uneven: the model-adjusted median onset-to-sampling delay was 4.8 days among 129 confirmed patients, while insecurity posed additional challenges for field response. Preparedness was further constrained by uncertainty about reservoir ecology, limited clinical validation of BDBV-specific diagnostics and the absence of MCMs with established efficacy in BVD. We therefore propose a One Health alert-to-action pathway linking human, animal, environmental and community signals to BDBV-inclusive confirmation, safe care, IPC, cross-border continuity, accountable governance and feedback-informed adaptation. Earlier activation is a preparedness gain, but protection depends on whether that advantage is carried through reliable, traceable and equitable operational hand-offs.

## Graphical abstract

**Figure:**
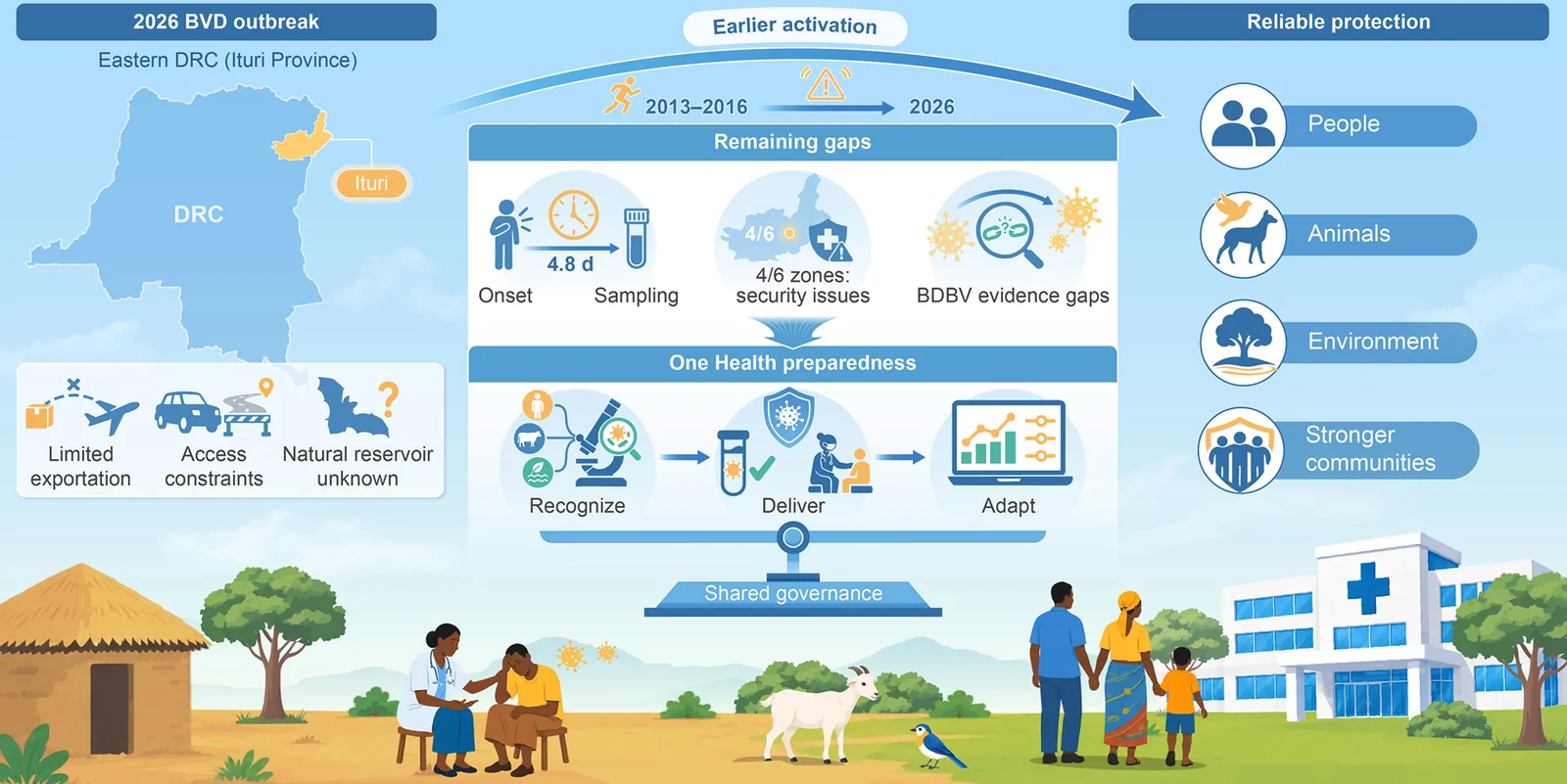

## 1 Introduction

The 2026 outbreak of Bundibugyo virus disease (BVD) in eastern Democratic Republic of the Congo (DRC) brought a long-standing preparedness gap into immediate focus [1,2]. BVD is caused by Bundibugyo virus (BDBV), first recognised during a large Ugandan outbreak in 2007 and later documented in the DRC [3,4]. Because few outbreaks have been recorded, direct laboratory and clinical experience remains limited [5]. No vaccine or therapeutic is licensed specifically for BVD, and candidate vaccines, monoclonal antibodies and antiviral drugs are being prioritised as medical countermeasures (MCMs) for clinical evaluation [6]. Preparedness must therefore distinguish transferable Ebola virus (EBOV)-based experience from questions requiring BDBV-specific evidence. Addressing these gaps also requires coordinated investigation and action across human, animal, laboratory, ecological and community systems.

The outbreak unfolded amid insecurity, population mobility and uneven access to affected health zones [2,7], with international exportation and limited secondary transmission outside the DRC [1,8]. Early international emergency decisions, coordination and MCM prioritisation widened the window for action [6,[9], [10], [11]]. The key test is whether this time advantage can be preserved through every stage: case recognition, sampling, laboratory result return, safe care and sustained community access. Because these hand-offs cross clinical, laboratory, public-health, community and cross-border systems, the outbreak tests whether those systems can function as a connected One Health pathway.

In this Review, One Health provides a practical framework for coordinating cross-sectoral action and linking early warning to field response. It begins with joint interpretation of human, animal, laboratory, ecological and community information [12]. Uncertain signals should prompt targeted verification without presuming an unconfirmed host or transmission pathway. BDBV-inclusive testing and confirmation should proceed alongside supportive care and infection prevention and control (IPC). Laboratory results should inform clinical and contact management, community communication and, where relevant, cross-border notification. Community feedback and operational records should then guide subsequent adaptation. One Health is thus treated as a connected alert-to-action pathway rather than as a set of parallel sectoral activities.

This structured narrative review synthesises peer-reviewed research, official outbreak reports, policy documents and operational guidance to examine whether earlier institutional activation was translated into reliable field delivery. It first defines the 2026 outbreak's scope and BDBV-specific evidence boundaries, then examines breakpoints in joint risk recognition, confirmation, safe care, community feedback and cross-border continuity. Finally, it proposes a pathway based on named responsibilities, predefined triggers, traceable hand-offs and feedback-led correction. Earlier activation is a preparedness gain, but its value depends on carrying the time gained through a BDBV-appropriate, traceable and adaptable One Health pathway.

## 2 Search strategy and selection criteria

We conducted a structured narrative review of evidence relevant to preparedness for and response to BVD. Literature searches were performed during manuscript development and were subsequently updated and standardised. The final database searches were completed on 12 August 2026, and epidemiological reporting was updated through 23 August 2026. Three database-adapted search families covered: (1) BVD/BDBV epidemiology, diagnostics and MCMs; (2) One Health, animal, reservoir, spillover and ecological surveillance; and (3) outbreak preparedness, conflict-affected access, community engagement, IPC and cross-border response. No database-level limits on publication date or document type were applied during retrieval; temporal, source-type and thematic criteria were applied during screening. After deduplication, records were screened by title and abstract and then assessed in full text using predefined criteria for temporal relevance, source type, thematic relevance, evidence function and transferability to BDBV (Fig. 1). Direct BDBV evidence was prioritised; evidence from outbreaks caused by other orthoebolaviruses was retained only when relevant and transferable. Supplementary searches used backward citation tracing, targeted searches by title or digital object identifier (DOI), and authoritative official sources to identify additional peer-reviewed, outbreak and policy evidence. Complete search strings, eligibility criteria, database-specific yields and additional sources are reported in Supplementary Table S3 and S4. Owing to heterogeneity in study designs and source types, evidence was synthesised narratively without meta-analysis or formal risk-of-bias assessment.

**Fig. 1:**
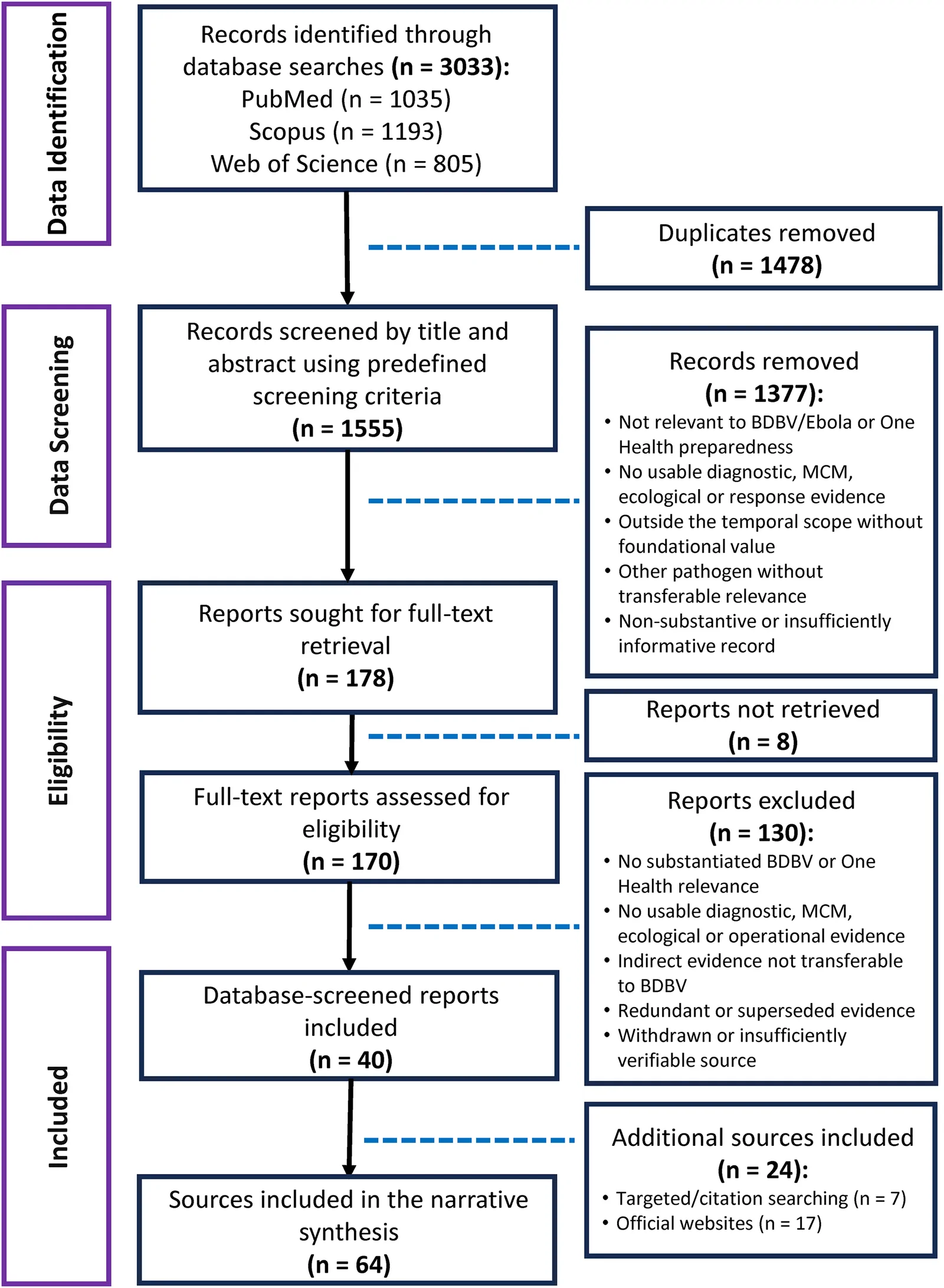
Identification and selection of sources for the structured narrative review. Note: BDBV, Bundibugyo virus; MCM, medical countermeasure.

## 3 Preparedness lessons from the 2026 BVD outbreak

### 3.1 Outbreak scope and cross-border spread

As of 23 August 2026, the DRC had reported 5584 confirmed BVD cases and 2680 confirmed deaths across 57 health zones in six provinces. Transmission remained concentrated in established hotspots in Ituri Province, intensified in parts of North Kivu Province and Haut-Uélé Province, and continued to expand geographically [13].

Cross-border impact remained limited relative to the burden in the DRC. Uganda had recorded 20 confirmed cases, including 15 imported infections and five secondary infections, while France had reported one imported case [13]. No sustained transmission had been documented outside the DRC. These exportations make cross-border continuity a necessary preparedness function [14], while the principal disease burden and field-delivery challenges remain in eastern DRC.

### 3.2 Institutional activation and field-delivery gaps

Fig. 2 compares the timing of selected, publicly documented institutional milestones during the opening phases of the 2013–2016 Ebola virus disease (EVD) epidemic in West Africa and the 2026 BVD outbreak. Both timelines use the date of the first World Health Organization (WHO) public report as day 0. For 2026, the comparison is limited to 16 May–27 June. It covers the determination of a public health emergency of international concern, the convening of the International Health Regulations (IHR) Emergency Committee, cross-border and continental coordination, response planning and early MCM prioritisation [6,[9], [10], [11],^15^]. By isolating this opening phase, the figure shows how quickly formal mechanisms created an action window before the outbreak's later evolution.

**Fig. 2:**
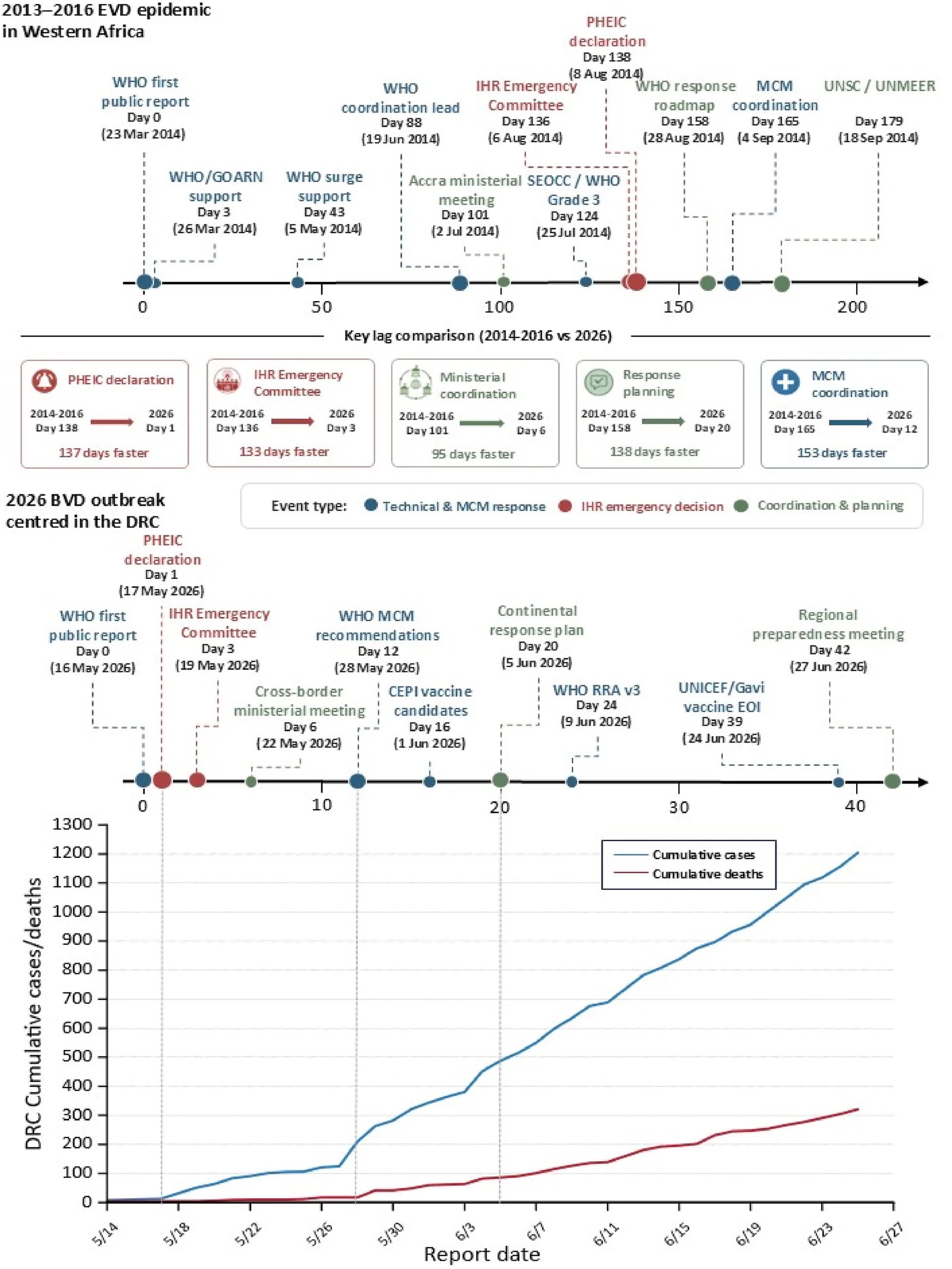
Early institutional response milestones and the corresponding initial epidemic curve during the 2013–2016 Ebola virus disease epidemic in West Africa and the 2026 BVD outbreak centred in the DRC. Note: Day 0 denotes the date of the first WHO public report. The comparison is restricted to publicly documented milestones in the opening response phase and does not assess subsequent field effectiveness. BVD, Bundibugyo virus disease; CEPI, Coalition for Epidemic Preparedness Innovations; DRC, Democratic Republic of the Congo; EOI, expression of interest; EVD, Ebola virus disease; GOARN, Global Outbreak Alert and Response Network; IHR, International Health Regulations; MCM, medical countermeasure; PHEIC, public health emergency of international concern; RRA, rapid risk assessment; SEOCC, Sub-Regional Ebola Operations and Coordination Centre; UNMEER, United Nations Mission for Ebola Emergency Response; UNSC, United Nations Security Council; WHO, World Health Organization.

Across broadly comparable milestones, key institutional mechanisms were activated earlier in 2026 than during the 2013–2016 epidemic (Fig. 2). This acceleration represents a meaningful preparedness gain because coordination, resource mobilisation, diagnostic deployment and research preparation could begin sooner. Fig. 2 therefore establishes the institutional opportunity against which downstream delivery can be examined.

Evidence from the 2026 outbreak nevertheless shows that the time gained through earlier institutional activation was not consistently translated into timely field action. Among 129 laboratory-confirmed patients with recorded symptom-onset and sampling dates, a model accounting for incomplete observation estimated a mean onset-to-sampling delay of 7.4 days and a median delay of 4.8 days [16]. This interval combines the time before presentation with the period from presentation through recognition to sampling; it therefore does not identify where the delay occurs. In a separate Bunia hospital cohort, 37 of 157 staff members were admitted with infectious symptoms. Ten were not tested, and multiple staff members had samples drawn late in their illness because initial samples or results were lost or testing teams arrived late [17]. Security conditions posed an additional operational challenge: among the six health zones in Ituri that had recorded confirmed cases as of 21 May 2026, four had recurrent security problems linked to land and resource disputes, ethnic tensions or armed-group activity [2]. Together, these findings show that case recognition, sampling and testing remained vulnerable to delay or interruption, while insecurity posed additional challenges for field response. Earlier activation widened the time available for action, but its public-health value depended on reliable hand-offs from alert to sampling, result return, care and response.

### 3.3 BDBV-specific confirmation and MCM readiness

Translating an alert into action requires diagnostic and MCM decisions grounded in evidence relevant to BDBV. During the 2007 outbreak, assays targeting EBOV and Sudan virus initially missed BDBV because of sequence divergence [3]. This experience supports BDBV-inclusive confirmation linked to standardised sampling, specimen referral and result return [[18], [19], [20], [21]].

Diagnostic readiness spans three evidence stages: regulatory access, analytical detection and validation in patients. In 2026, WHO listing of the first BDBV molecular diagnostic advanced regulatory access [22]. A pan-filovirus reverse-transcription quantitative polymerase chain reaction (RT-qPCR) assay and an investigational pan-orthoebolavirus lateral-flow assay also detected BDBV material [23,24], extending the available analytical options. Their evaluations used EBOV clinical samples or laboratory and animal material, so performance in patients with BVD remains to be established. Field readiness additionally requires integration into specimen-referral and result-return networks.

No vaccine or therapeutic is licensed for BVD [6], so MCM readiness must distinguish transferable EBOV evidence from BDBV-specific preclinical and clinical evidence. In EBOV trials, ring vaccination was efficacious [25], and mAb114 and REGN-EB3 improved 28-day survival relative to ZMapp [26]. Earlier admission and lower viral load predicted better outcomes across groups in the Pamoja Tulinde Maisha trial [26]. One EBOV vaccination study found predominantly EBOV-specific responses with limited cross-reactivity [27], whereas another detected BDBV-binding antibodies of uncertain protective significance [28]. Other BDBV-relevant evidence includes remdesivir activity in cell culture [29] and protection by antibody and vaccine candidates in nonhuman primates [[30], [31], [32], [33]].

Obeldesivir evidence comes from an EBOV nonhuman-primate model [34]. Prioritisation by WHO and the Coalition for Epidemic Preparedness Innovations (CEPI), together with enrolment in the Platform Adaptive Randomised Trial for New and Repurposed Filovirus Treatments (PARTNERS), shows that candidates are entering organised evaluation for BVD [6,35,36]. Current care and control therefore continue to rely on timely confirmation, supportive care and IPC [37]. From a One Health perspective, readiness requires that each tool be matched to its evidence stage and integrated into a coordinated pathway linking detection, care, response and research.

## 4 One Health alert-to-action gaps

One Health preparedness depends not simply on whether individual capacities exist, but on whether the output of one stage becomes a usable input for the next. Delays before sampling, security-related operational challenges and cross-border care-seeking during the 2026 outbreak illustrate threats to continuity at operational hand-offs [2,8,16,38]. The following sections trace the alert-to-action pathway from multisource risk recognition to confirmation and safe care. They then examine whether community feedback reshapes service delivery and whether care and surveillance remain connected across institutions and borders. Table 1 summarises the preparedness capacities, representative measures and evidence maturity supporting these processes.

**Table 1:** One Health preparedness for Bundibugyo virus disease: evidence-informed operational functions and evidence maturity.

Preparedness domain	Representative tools or measures and operational role	BDBV relevance and evidence maturity
Joint human–animal–environmental risk recognition	Clinical and community alerts; unusual animal morbidity or mortality reports; risk-based animal or wildlife sampling and molecular or serological testing; exposure and ecological information; and cross-sector interpretation. These inputs trigger targeted investigation without prespecifying a reservoir.	Transferable implementation and BDBV research-stage evidence. One Health principles and spillover frameworks support integrated interpretation [12,39], and DRC studies illustrate joint investigation and traceable workflows [40,41]. Experimental swine susceptibility does not establish a natural reservoir or spillover pathway [42]. Transferable wildlife-surveillance evidence identifies implementation gaps relevant to animal and ecological signal collection [43].
BDBV-inclusive confirmation and specimen referral	Broad filovirus screening; BDBV-specific nucleic acid testing; sequencing; and standardised collection, referral and result return. These measures reduce species-related diagnostic blind spots and connect confirmation to action.	Current WHO guidance supports standardised specimen collection, diagnostic analysis and result reporting [21]. BDBV-relevant evidence remains at different stages of validation: Historical identification and the 2026 WHO emergency use listing support BDBV-inclusive confirmation [3,22]. Broad platforms have analytical or non-patient BDBV evidence, while patient-level field performance remains incompletely established [23,24].
Safe care, IPC and workforce protection	Syndromic triage, appropriate separation, safe sampling and transport, supportive care, IPC, personal protective equipment, exposure management and referral. These actions should begin at the suspected-case stage and span primary, private and referral facilities.	Established operational principles for filovirus response. WHO guidance supports early care and IPC [37]. EBOV studies identify multi-facility exposure, facility-level implementation support and changes observed after reinforcement [[44], [45], [46]]; these data inform implementation design rather than measure effects during the 2026 BVD outbreak.
Community-based surveillance and operational feedback	Community alerts and death reporting; referral follow-up; risk communication and community engagement; structured feedback; and action tracking. These mechanisms connect local concerns with investigation, service adjustment and visible follow-up.	Transferable DRC implementation evidence. WHO guidance defines the operational roles of risk communication, community engagement and structured feedback [47], while DRC studies link trust, feedback uptake and community surveillance with care-seeking, operational adjustment and referral [[48], [49], [50]]. The evidence supports these functions without estimating their causal effect on transmission or mortality.
Cross-border coordination and continuity	Compatible case and exposure definitions; cross-border notification and referral; shared specimen documentation, laboratory results and contact information; and predefined backup laboratory, transport, communication and referral routes. These measures extend preparedness across the public and private facilities used by mobile populations.	Operational checklists define transferable institutional interfaces for imported-case preparedness [51]. Documented 2026 importations provide direct event evidence of multimodal travel and private-facility-to-laboratory hand-offs [8,38], while security conditions and 2024 facility-readiness data provide context for operational challenges and capacity gaps [2,52].
MCM and research readiness	BDBV-targeted vaccines; broader antibody and antiviral candidates; adaptive or master protocols; advance ethics, regulatory, data and sample arrangements; trained sites; standard supportive care; community governance; and access planning. Together, these components enable stage-appropriate evaluation.	Research-stage evidence. No MCM has established clinical efficacy for BVD. Available evidence comprises EBOV trials, BDBV cell and animal studies and candidate prioritisation [6,25,26,29,30,32,33]. PARTNERS enrolment indicates that evaluation has begun, not that efficacy has been established [36].

Abbreviations: BDBV, Bundibugyo virus; DRC, Democratic Republic of the Congo; EBOV, Ebola virus; IPC, infection prevention and control; MCM, medical countermeasure; PARTNERS, Platform Adaptive Randomised Trial for New and Repurposed Filovirus Treatments; WHO, World Health Organization.

### 4.1 Joint risk recognition under reservoir uncertainty

The natural reservoir of BDBV and its route into humans remain unconfirmed. Animal, ecological, laboratory and human-exposure signals should therefore inform joint risk assessment rather than premature host attribution. Zoonotic spillover arises from interactions among ecological conditions, host characteristics, exposure opportunities and human susceptibility, not from a single signal [39]. In a controlled experimental infection study, BDBV replicated in domestic pigs and was shed through oral and nasal routes, although most infected pigs showed no obvious clinical illness [42]. This finding suggests that surveillance focused only on visibly ill animals could miss risk signals from experimentally susceptible animals. Field investigation also requires signals from different sources to be interpreted together. An EBOV-centred One Health investigation in Tshuapa Province combined wildlife PCR screening, animal and human serology, and interviews on wildlife contact. No EBOV RNA was detected in 1049 animal samples. Orthoebolavirus-reactive immunoglobulin G (IgG) signals were detected in selected bat, rodent and human samples, while interviews documented frequent forest visits and wildlife hunting [40]. Given serological cross-reactivity among ebolaviruses [53], these findings helped identify animal groups, exposure settings and populations for further investigation but did not identify a natural reservoir.

Turning these separate observations into a joint risk assessment requires surveillance infrastructure for the systematic collection, documentation and analysis of animal-health signals [43]. A broader zoonotic surveillance initiative in nine DRC provinces connected local veterinarians, community animal-health workers, field-sampling and laboratory teams through traceable specimens and structured records [41]. Such records retain the source, species, location and evidentiary status of each signal, allowing participating sectors to decide whether further sampling, human-exposure assessment or escalation is warranted [12]. In this setting, One Health provides a way to convert dispersed observations into a proportionate and traceable investigation without presuming an unconfirmed host.

### 4.2 Linking BDBV confirmation with safe care

Safe management of a suspected BVD case should begin while confirmation is pending, with appropriate separation, IPC and supportive care adjusted as results become available [37,54]. The first recognised BVD outbreak in Uganda in 2007 showed that completing this process requires sensitive case detection, standardised specimen collection, safe packaging, reliable transport and specific laboratory confirmation [21,55]. Evidence from the 2026 outbreak further indicates that case recognition and sampling can still create a substantial front-end delay [16]. Once confirmation is available, the result must reach the facility responsible for the patient's care and the relevant public-health teams so that they can confirm or adjust isolation, referral, supportive care and contact management [37].

Safe care must extend across the ordinary public and private facilities through which patients move, rather than beginning only at specialist treatment centres. During the 2018–2020 DRC outbreak, 579 of 3481 EVD cases (16.6%) were classified as nosocomial. Among patient or visitor nosocomial cases with traceable care-seeking histories, 84.5% had attended at least two facilities before detection or transfer, and half of the facilities associated with nosocomial infection were privately owned [44]. These findings show how unrecognised patients can move through first-contact facilities and create opportunities for healthcare-associated transmission before specialist referral. These facilities therefore need not only to participate in case-detection and referral arrangements, but also to manage suspected cases safely before transfer. Building this capacity requires training, supplies and on-site guidance. A North Kivu study evaluated this implementation approach. Forty-five trained local medical students served as mentors across 101 high-risk facilities, supporting healthcare workers in IPC, supply management and active case finding. Facilities receiving this combined support were more likely to achieve the prespecified IPC threshold [46]. The study therefore provides an operational example of how mentorship, training, supply management, on-site problem solving and continued monitoring can extend IPC capacity to facilities that may receive patients before specialist referral.

### 4.3 Community feedback for adaptive delivery

Community feedback can support outbreak response by helping residents enter formal care and by revealing how services need to change [47]. In a 2018 survey of 961 adults in Beni and Butembo, only 31.9% believed that local authorities represented their interests. Lower institutional trust and belief in misinformation were also associated with less formal care-seeking and lower acceptance of EVD vaccination [48]. These associations link trust to entry into formal response pathways. During the 2018–2020 outbreak, a Red Cross system collected approximately 300,000 verbatim feedback records across 29 health zones, with some concerns prompting changes such as body bags with viewing windows and greater family participation in safe burials [49]. In two Ituri health areas, community health workers reported ill community members for investigation by response teams. Suspected cases were referred to an integrated treatment centre for isolation and testing, while other referred patients received primary care. Overall, 73.1% of 6711 community referrals reached the centre [50]. These practices connect residents to investigation and referral and create a route for community concerns to inform service design. Yet feedback changes the response only when it is interpreted and incorporated into decisions.

The 2018–2020 North Kivu outbreak shows what can happen when this final step is missed. Some residents interpreted the government-led response, military escorts and links between response institutions and state authorities through the lens of earlier violence, political exclusion and electoral disenfranchisement. Residents' concerns extended beyond limited disease knowledge to the safety and trustworthiness of these arrangements and the perceived ties between response institutions and parties to the conflict. A separate analysis of six Red Cross feedback reports found that these concerns had been recorded but were often framed mainly as rumours or misinformation. The associated recommendations emphasised further disease education and explanations of treatment, with less attention to whether security arrangements or institutional relationships themselves required adaptation [56]. In the same outbreak, an integrated analytics unit followed a different pathway. It combined social and behavioural findings with epidemiological and health-service data and generated 112 recommendations from 58 studies. Full or partial implementation was recorded for 86% of these recommendations [57]. This contrast identifies the operational step that must follow listening: community observations need to be translated into explicit recommendations, and their implementation needs to be tracked.

### 4.4 Cross-border continuity for equitable access

Infected people may cross borders before BVD is recognised or confirmed. Cross-border continuity therefore requires continuous links among case recognition, specimen referral, result communication, clinical referral and the exchange of contact information across countries and institutions. Two documented cases imported into Uganda from Ituri Province first presented to separate private hospitals in Kampala [8]. After one patient died, mortality surveillance triggered specimen referral; the national laboratory confirmed BDBV and reported the result to the hospital and Ministry of Health [38]. This sequence shows that identifying a cross-border patient can depend on a continuous chain of hand-offs linking receiving facilities, mortality surveillance, specimen transport, a national laboratory and public-health authorities. Extending this chain across countries requires compatible case and exposure definitions, referral protocols, specimen documentation, result exchange and contact information [10,51].

To keep these hand-offs continuous, preparedness must cover the full range of facilities that cross-border patients actually attend, rather than concentrating only on designated public facilities in border districts. A 2024 survey of 214 public facilities in southwestern Ugandan border districts found that 47.2% had basic isolation units, 54.2% had trained laboratory personnel and 33.6% achieved high case-management readiness [52]. These findings indicate uneven baseline capacity to connect patients to isolation, confirmation and case management within the surveyed public network. The documented Kampala private hospitals fell outside the survey's scope because it covered a different provider type and geographical area [8,38]. The survey therefore could not assess whether these actual first-contact facilities were prepared to recognise, isolate, sample, notify and refer a suspected case. If such entry points are not covered by preparedness assessment, IPC support, notification and referral arrangements, delays in these functions may interrupt the pathway at first contact. Preparedness planning should therefore follow real care-seeking routes and encompass public and private providers from border areas to urban referral centres. When every entry point can connect patients to the same pathway for confirmation, supportive care and public-health follow-up, access to essential services becomes less dependent on the type or location of the first facility attended [58,59].

## 5 Operationalising the alert-to-action pathway

The operational requirements above form a functioning alert-to-action pathway only when decision-making, implementation and feedback operate as a continuous cycle (Fig. 3). Shared governance assigns responsibility and defines when action should escalate. Traceable implementation shows whether each hand-off is completed, while structured feedback identifies recurrent failures, guides adaptation and reveals evidence gaps requiring further study.

**Fig. 3:**
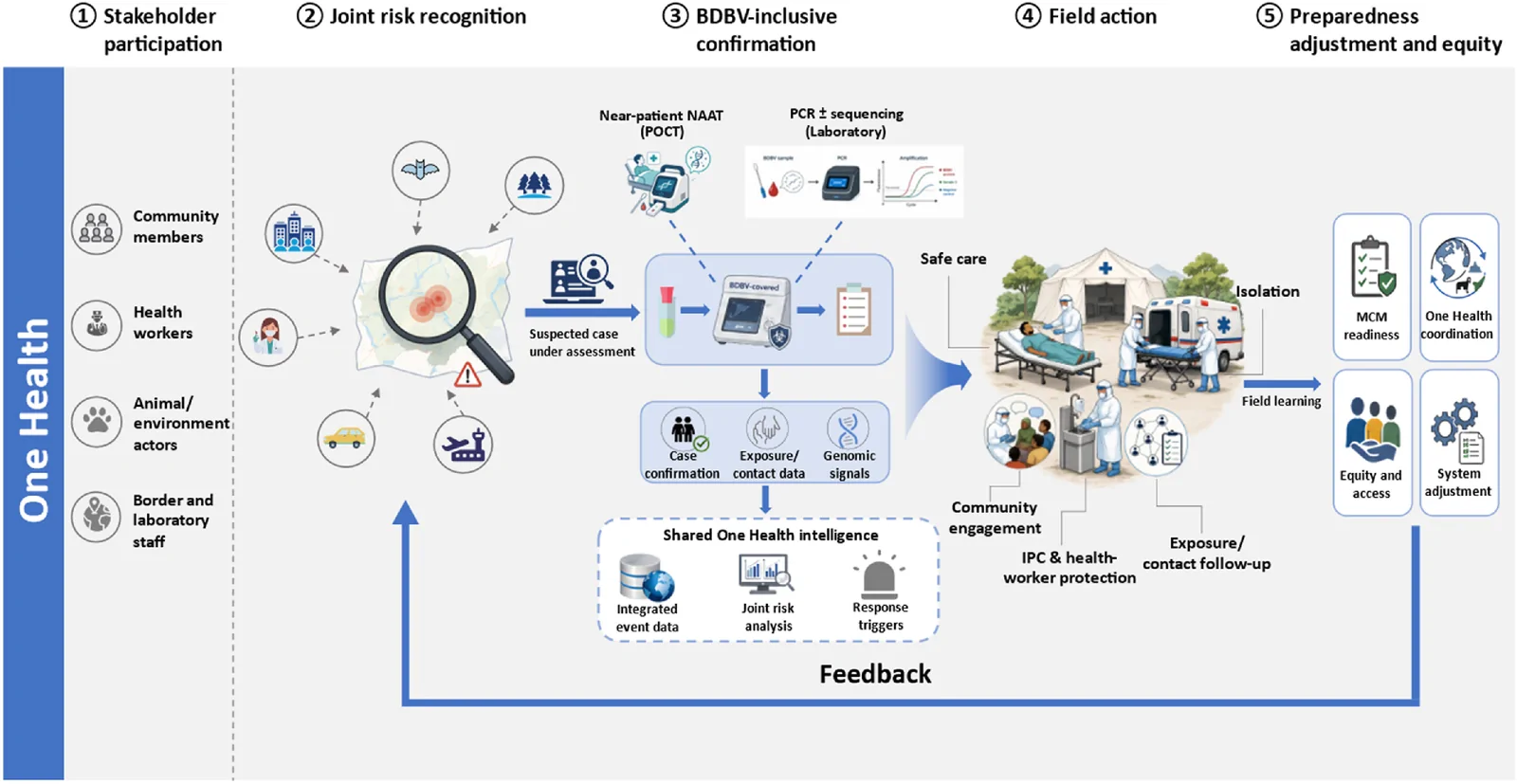
One Health preparedness pathway from multisource alert to field action, feedback and adaptation for BVD. Note: The pathway is conceptual and does not imply that all components were fully implemented during the 2026 outbreak. BDBV, Bundibugyo virus; IPC, infection prevention and control; MCM, medical countermeasure; NAAT, nucleic acid amplification test; PCR, polymerase chain reaction; POCT, point-of-care testing. Selected illustrative elements in Fig. 3 were generated using ChatGPT Images 2.0 (OpenAI) and subsequently edited and assembled by the authors.

### 5.1 Shared governance for accountable decisions

One Health alerts may originate from community, clinical, animal-health, environmental and laboratory systems, so no single team can verify every signal and authorise the full response [60,61]. Shared governance is therefore needed to specify who receives and verifies alerts, how evidence is combined, who authorises escalation and who tracks subsequent action. In Ghana, collaboration remained reactive when detection, analysis and data management stayed within sectoral silos [62]. Community and clinical teams identify compatible illness and exposure; animal-health and environmental teams contribute unusual-event and ecological information; laboratories characterise signals; and a coordinating body synthesises evidence, records decisions and tracks actions [12]. Traceable specimens and structured records can connect field and laboratory actors to this decision pathway [41].

Participating sectors should agree escalation rules in advance. These rules should define when an alert requires verification, when verified findings warrant joint investigation and when the response should be expanded. Severe illness compatible with BVD, unusual clusters, animal or environmental anomalies and cross-border exposure are examples of signals that should prompt verification. Decisions to initiate a joint investigation or expand the response should reflect the strength of the verified signal and its potential consequences, without presuming an unconfirmed host or transmission route [10,11]. Each alert record should include its source, named lead, status, rationale and assigned actions.

### 5.2 Traceable implementation and process indicators

Once a patient meets the suspected-case definition, confirmatory testing and protective action should proceed in parallel. While testing is pending, safe sampling, appropriate separation, supportive care and IPC should begin, and the public-health team should be notified [37]. When the BDBV result is available, the laboratory should communicate it to the responsible clinical and public-health teams and confirm receipt. Those teams should record the resulting decisions and whether isolation, referral, contact management, community communication and, where appropriate, cross-border notification were completed.

For operational review, we propose four separate intervals: investigation to sampling, sampling to laboratory receipt, receipt to result, and result communication to action. During the 2026 Ugandan importation, the national laboratory reported the RT-qPCR result to the hospital and Ministry of Health within 5 h of receiving the specimen [38]. This interval covers only laboratory receipt to result communication, not earlier recognition and transport or subsequent action. Recording these intervals together with the completion status of referral and notification [10] allows delays to be identified more precisely than with a single end-to-end measure. Backup laboratory, transport, communication and referral arrangements should be defined before normal routes are disrupted. Together, timing and completion records show where a hand-off failed, which team was responsible and whether subsequent correction improved performance.

### 5.3 Feedback-driven correction and evidence generation

Traceable records should not serve merely as an archive of actions. Their practical value lies in helping response teams identify operational failures, initiate corrective action and determine at a subsequent review whether the problem has been resolved. Joint review of community feedback, service data, access constraints and hand-off records can identify recurrent delays, coverage gaps and incomplete actions. Each problem should be assigned to a responsible team, with a corrective action and deadline; the next review should assess completion and recurrence [49,57]. A separate analysis of a North Kivu IPC support initiative examined 92 private primary-care facilities. Median IPC checklist scores were 19/36 at baseline, 30/36 after a four-month package of supplies, training and on-site mentorship, and 28/36 six months later [45]. This baseline–support–reassessment sequence makes change and subsequent drift visible and provides a basis for deciding whether reinforcement is needed.

Some evidence gaps cannot be resolved by refining service delivery alone and require direct clinical evidence generated during outbreaks. Because BVD outbreaks may provide only a short enrolment window, core trial arrangements should be prepared in advance [63,64]. These include master protocols, BDBV confirmation criteria, standard supportive care, ethical and regulatory procedures, data and sample governance, trained sites and independent oversight. On 2 July 2026, PARTNERS began enrolling patients with laboratory-confirmed BVD in the DRC into a randomised controlled platform trial to determine whether candidate therapeutics improve survival [36]. The trial links laboratory confirmation, standard supportive care, therapeutic evaluation and independent safety oversight in one operational framework. It shows how advance preparation can enable rigorous evidence generation during an outbreak response. The resulting evidence can inform future candidate selection and response decisions. Institutions and communities in affected regions should also participate in research governance and share in the durable capacity and benefits generated [58,59].

## 6 Conclusions

The 2026 BVD outbreak shows that faster institutional mobilisation is necessary but insufficient for effective preparedness. Its value depends on whether early decisions are carried through a connected One Health pathway. That pathway must link multisource risk recognition, BDBV-inclusive confirmation, safe care and IPC, feedback-informed adaptation, and continuity across institutions and borders. It must also account for unresolved reservoir ecology and the different evidence stages of diagnostics and MCMs. Clear responsibilities, predefined triggers, traceable hand-offs and substantive participation by affected institutions and communities can make failures visible, guide adaptation and support equitable access. Preparedness should therefore be judged not only by how quickly systems activate, but by whether they convert that early advantage into reliable protection.

## CRediT authorship contribution statement

**Yuxuan Shao:** Writing – original draft, Visualization, Methodology, Investigation, Conceptualization. **Jue Liu:** Writing – review & editing, Supervision, Methodology. **Lei Zhou:** Writing – review & editing, Supervision, Conceptualization.

## Ethics statement

Ethics approval and informed consent were not required because this review was based on published literature and publicly available sources and did not involve primary research with human participants or animals. Consent for publication was not applicable.

## Data availability statement

No new primary data were generated for this review. The evidence reviewed is available in the cited publications and official sources. Source information and data underlying Fig. 2, database search strategies, and evidence-selection criteria are provided in the Supplementary Appendix.

## Declaration of generative AI and AI-assisted technologies in the manuscript preparation process

During the preparation of this work, the authors used ChatGPT (OpenAI) to assist with translation, language editing, content organisation, and reference and citation checking. Selected illustrative elements in Fig. 3 were generated using ChatGPT Images 2.0 (OpenAI). The authors reviewed and revised the AI-assisted content and take full responsibility for the content of the publication.

## Funding

This work was supported by the Prevention and Control of Emerging and Major Infectious Diseases—National Science and Technology Major Project (grant number 2025ZD01900800) and the Public Health Professional Training Supporting Project (grant number 01062). The funders had no role in study design, data collection, data analysis, interpretation of results, manuscript preparation or the decision to submit the manuscript for publication.

## Declaration of competing interest

Lei Zhou serves as Editorial Board Member of *Science in One Health*. She was not involved in the peer review, editorial handing, or decision-making process for this manuscript and had no access to information regarding its peer review. Full responsibility for the editorial process for this manuscript was delegated to another journal editor. The authors declare no other competing interests.
