# Supplementary material for: One Health preparedness for Ebola disease caused by Bundibugyo virus: lessons from the 2026 outbreak centred in the Democratic Republic of the Congo
Source: Sci One Health. 2026 Sep 29;5:100170. doi: 10.1016/j.soh.2026.100170 (PMC13694988; doi:10.1016/j.soh.2026.100170)
Supplement: Supplementary file 1 [file mmc1.docx]

**Supplementary appendix**

**Supplementary Methods. Literature search and evidence selection**

This structured narrative review examined evidence relevant to preparedness for and response to Bundibugyo virus disease (BVD). PubMed, Scopus and Web of Science Core Collection were searched on 12 August 2026 using three complementary, database-adapted search families. N1 covered BVD and Bundibugyo virus (BDBV) epidemiology, virology, diagnostics and medical countermeasures (MCMs); N2 covered One Health, animal, reservoir, spillover and ecological surveillance; and N3 covered outbreak preparedness, conflict-affected access, community engagement, infection prevention and control (IPC), and cross-border response. No date, publication-type, language, full-text or subject-area filters were applied at retrieval. Complete search strategies and database-specific yields are reported in Table S3.

The nine searches yielded 3,033 records (PubMed, 1,035; Scopus, 1,193; Web of Science Core Collection, 805). Records were deduplicated sequentially by normalised digital object identifier (DOI), PubMed identifier (PMID), database accession identifier and, when identifiers were incomplete, conservative normalised-title and first-author matching. Conflicting identifiers and borderline title matches were not merged automatically. Deduplication removed 1,478 occurrences, leaving 1,555 unique records. Title and abstract screening used predefined criteria for temporal relevance, source type, thematic and evidentiary relevance, and transferability to BDBV. Full text was sought for 178 reports; eight could not be retrieved, and 170 were assessed. Forty database-screened reports were retained in the final narrative synthesis. The 130 reports not retained included sources that did not meet the defined evidence-function or transferability requirements, whose relevant content could not be verified, or whose contribution was redundant or superseded by stronger evidence.

Database screening was supplemented by backward citation tracing and targeted title, topic or DOI searches for peer-reviewed evidence, and by targeted searches of authoritative institutional websites for current outbreak reports, operational guidance, regulatory updates and MCM status. All additional sources were assessed against the same relevance, verifiability, transferability and non-redundancy criteria. Seven additional peer-reviewed reports and 17 official or institutional web sources were included, yielding 64 unique sources in the narrative synthesis (Figure 1; Table S4). Evidence was organised by function across BVD/BDBV, One Health risk recognition, confirmation, safe care, community engagement, cross-border continuity and preparedness implementation. Given the heterogeneity of source types and study designs, findings were synthesised narratively; no meta-analysis or formal risk-of-bias score was applied.

**Table S1 lists the publicly available source documents used to identify the event nodes shown in Figure 2.** Table S1. Source documents for event nodes in Figure 2

| **Outbreak** | **Event node** | **Source URL** |
| --- | --- | --- |
| 2013–2016 EVD epidemic in Western Africa | WHO first public report | https://www.who.int/emergencies/disease-outbreak-news/item/2014_03_23_ebola-en |
|  | WHO/GOARN support | https://www.who.int/emergencies/disease-outbreak-news/item/2014_03_26_ebola-en |
|  | WHO surge support | https://www.who.int/emergencies/disease-outbreak-news/item/2014_05_06_ebola-en |
|  | WHO coordination lead | https://www.who.int/news-room/spotlight/one-year-into-the-ebola-epidemic/key-events-in-the-who-response-to-the-ebola-outbreak |
|  | Accra ministerial meeting | https://www.who.int/news-room/spotlight/one-year-into-the-ebola-epidemic/key-events-in-the-who-response-to-the-ebola-outbreak |
|  | SEOCC / WHO Grade 3 | https://www.who.int/news-room/spotlight/one-year-into-the-ebola-epidemic/key-events-in-the-who-response-to-the-ebola-outbreak |
|  | IHR Emergency Committee | https://www.who.int/news/item/08-08-2014-statement-on-the-1st-meeting-of-the-ihr-emergency-committee-on-the-2014-ebola-outbreak-in-west-africa |
|  | PHEIC declaration | https://www.who.int/news/item/08-08-2014-statement-on-the-1st-meeting-of-the-ihr-emergency-committee-on-the-2014-ebola-outbreak-in-west-africa |
|  | WHO response roadmap | https://iris.who.int/bitstream/handle/10665/131596/EbolaResponseRoadmap.pdf |
|  | MCM coordination | https://www.who.int/publications/i/item/WHO-EVD-Meet-EMP-14.1 |
|  | UNSC / UNMEER | https://digitallibrary.un.org/record/779813?ln=en |
| 2026 BVD outbreak centred in the DRC | WHO first public report | https://www.who.int/emergencies/disease-outbreak-news/item/2026-DON602 |
|  | PHEIC declaration | https://www.who.int/news/item/17-05-2026-epidemic-of-ebola-disease-in-the-democratic-republic-of-the-congo-and-uganda-determined-a-public-health-emergency-of-international-concern |
|  | IHR Emergency Committee | https://www.who.int/news/item/22-05-2026-first-meeting-of-the-ihr-emergency-committee-regarding-the-epidemic-of-ebola-bundibugyo-virus-disease-in-the-democratic-republic-of-the-congo-and-uganda-2026-temporary-recommendations |
|  | Cross-border ministerial meeting | https://www.afro.who.int/news/high-level-ministerial-meeting-cross-border-coordination-ebola-disease-outbreak-caused |
|  | WHO MCM recommendations | https://www.who.int/news/item/28-05-2026-experts-convened-by-who-advise-on-candidate-treatments-and-vaccines-for-ebola-disease-caused-by-bundibugyo-virus |
|  | CEPI vaccine candidates | https://cepi.net/cepi-fast-tracks-three-bundibugyo-ebolavirus-vaccine-candidates |
|  | Continental response plan | https://www.who.int/news/item/05-06-2026-africa-cdc-and-who-launch-joint-continental-ebola-response-plan |
|  | WHO RRA v3 | https://www.who.int/publications/m/item/who-rapid-risk-assessment-ebola-disease-caused-by-bundibugyo-virus--democratic-republic-of-the-congo--uganda-and-countries-with-land-borders-adjoining-countries-with-documented-bdbv-detection-v3 |
|  | UNICEF/Gavi vaccine EOI | https://www.unicef.org/press-releases/unicef-and-gavi-launch-call-manufacturers-support-accelerated-access-bundibugyo |
|  | Regional preparedness meeting | https://www.afro.who.int/fr/node/23445 |

**Table S2 provides the reporting-date cumulative case and death data used to plot the DRC epidemic curve in Figure 2; data were extracted from publicly available INSP/COUSP outbreak updates and situation reports (source website: https://insp.cd/blog-2/).**

Table S2. Source data for the DRC epidemic curve in Figure 2

| Reporting date | Cumulative cases | Cumulative deaths |
| --- | --- | --- |
| 2026-05-14 | 8 | 4 |
| 2026-05-17 | 13 | 4 |
| 2026-05-19 | 33 | 4 |
| 2026-05-19 | 51 | 4 |
| 2026-05-20 | 64 | 6 |
| 2026-05-21 | 83 | 9 |
| 2026-05-22 | 91 | 10 |
| 2026-05-23 | 101 | 10 |
| 2026-05-24 | 105 | 10 |
| 2026-05-25 | 106 | 12 |
| 2026-05-26 | 121 | 17 |
| 2026-05-27 | 125 | 17 |
| 2026-05-28 | 210 | 17 |
| 2026-05-29 | 263 | 42 |
| 2026-05-30 | 282 | 42 |
| 2026-05-31 | 321 | 48 |
| 2026-06-01 | 344 | 60 |
| 2026-06-02 | 363 | 62 |
| 2026-06-03 | 381 | 64 |
| 2026-06-04 | 452 | 82 |
| 2026-06-05 | 488 | 86 |
| 2026-06-06 | 515 | 91 |
| 2026-06-07 | 550 | 101 |
| 2026-06-08 | 598 | 115 |
| 2026-06-09 | 635 | 127 |
| 2026-06-10 | 676 | 136 |
| 2026-06-11 | 689 | 139 |
| 2026-06-13 | 782 | 181 |
| 2026-06-14 | 808 | 192 |
| 2026-06-15 | 837 | 196 |
| 2026-06-16 | 875 | 202 |
| 2026-06-17 | 896 | 232 |
| 2026-06-18 | 933 | 245 |
| 2026-06-19 | 956 | 247 |
| 2026-06-20 | 1003 | 254 |
| 2026-06-21 | 1048 | 267 |
| 2026-06-22 | 1094 | 277 |
| 2026-06-23 | 1118 | 291 |
| 2026-06-24 | 1155 | 304 |
| 2026-06-25 | 1203 | 321 |

**Table S3 reports the complete database-specific strategies used in the structured narrative review, together with the date and number of records exported from each search.**

Table S3. Database search strategies used for the structured narrative review

| **Search ID** | **Database** | **Evidence domain** | **Search date** | **Complete search strategy** | **Records retrieved** |
| --- | --- | --- | --- | --- | --- |
| N1-PM | PubMed | BVD/BDBV epidemiology, virology, diagnostics and MCMs | 12 Aug 2026 | (  "Bundibugyo virus"[Title/Abstract] OR "Bundibugyo ebolavirus"[Title/Abstract]  OR "Bundibugyo Ebola virus"[Title/Abstract] OR "Bundibugyo virus disease"[Title/Abstract]  OR "Orthoebolavirus bundibugyoense"[Title/Abstract]  OR (BDBV[Title/Abstract] AND (Ebola[Title/Abstract] OR ebolavirus[Title/Abstract] OR orthoebolavirus[Title/Abstract]))  ) | 222 |
| N2-PM | PubMed | One Health, animal, reservoir, spillover and ecological surveillance | 12 Aug 2026 | (  ((Ebola[Title] OR ebolavirus*[Title] OR orthoebolavirus*[Title] OR "Bundibugyo virus"[Title/Abstract] OR BDBV[Title/Abstract])  AND (reservoir*[Title] OR "natural host"[Title/Abstract] OR "natural hosts"[Title/Abstract]  OR "animal host"[Title/Abstract] OR "animal hosts"[Title/Abstract] OR bat[Title] OR bats[Title]  OR wildlife[Title] OR zoono*[Title] OR spillover*[Title] OR "cross-species transmission"[Title/Abstract]  OR "animal surveillance"[Title/Abstract] OR "wildlife surveillance"[Title/Abstract]  OR "environmental surveillance"[Title/Abstract] OR ecolog*[Title]))  OR ("One Health"[Title] AND ("early warning"[Title] OR "early detection"[Title] OR surveillance[Title])  AND (zoono*[Title/Abstract] OR outbreak*[Title/Abstract]))  OR ("One Health"[Title] AND (definition[Title] OR "conceptual framework"[Title] OR "operational framework"[Title]))  OR (zoono*[Title] AND spillover*[Title] AND (pathway*[Title] OR framework*[Title] OR driver*[Title]))  ) | 342 |
| N3-PM | PubMed | Preparedness, conflict-affected access, community engagement, IPC and cross-border response | 12 Aug 2026 | (  Ebola[Title] OR ebolavirus*[Title] OR orthoebolavirus*[Title]  OR "Bundibugyo virus"[Title/Abstract] OR BDBV[Title/Abstract]  )  AND  (  ("outbreak preparedness"[Title/Abstract] OR "outbreak response"[Title/Abstract]  OR "humanitarian emergency"[Title/Abstract] OR "armed conflict"[Title/Abstract])  OR ("community engagement"[Title/Abstract] OR mistrust[Title/Abstract]  OR "risk communication"[Title/Abstract] OR "infection prevention and control"[Title/Abstract])  OR ("cross-border"[Title/Abstract] OR "introduction risk"[Title/Abstract] OR "health equity"[Title/Abstract])  ) | 471 |
| N1-SC | Scopus | BVD/BDBV epidemiology, virology, diagnostics and MCMs | 12 Aug 2026 | TITLE-ABS-KEY(  "Bundibugyo virus" OR "Bundibugyo ebolavirus" OR "Bundibugyo Ebola virus"  OR "Bundibugyo virus disease" OR "Orthoebolavirus bundibugyoense"  OR (BDBV AND (Ebola OR ebolavirus OR orthoebolavirus))  ) | 278 |
| N2-SC | Scopus | One Health, animal, reservoir, spillover and ecological surveillance | 12 Aug 2026 | TITLE(Ebola OR ebolavirus* OR orthoebolavirus* OR "Bundibugyo virus" OR BDBV)  AND ABS(reservoir* OR "natural host" OR "animal host" OR bat OR wildlife OR zoono* OR spillover*  OR "cross-species transmission" OR "animal surveillance" OR "wildlife surveillance"  OR "environmental surveillance") | 473 |
| N3-SC | Scopus | Preparedness, conflict-affected access, community engagement, IPC and cross-border response | 12 Aug 2026 | TITLE(Ebola OR ebolavirus* OR orthoebolavirus* OR "Bundibugyo virus" OR BDBV)  AND ABS("outbreak preparedness" OR "outbreak response" OR "humanitarian emergency"  OR "armed conflict" OR "community engagement" OR mistrust OR "risk communication"  OR "infection prevention and control" OR "cross-border" OR "introduction risk" OR "health equity") | 442 |
| N1-WO | Web of Science Core Collection | BVD/BDBV epidemiology, virology, diagnostics and MCMs | 12 Aug 2026 | TI=("Bundibugyo virus" OR "Bundibugyo ebolavirus" OR "Bundibugyo Ebola virus"  OR "Bundibugyo virus disease" OR "Orthoebolavirus bundibugyoense"  OR (BDBV AND (Ebola OR ebolavirus OR orthoebolavirus)))  OR AB=("Bundibugyo virus" OR "Bundibugyo ebolavirus" OR "Bundibugyo Ebola virus"  OR "Bundibugyo virus disease" OR "Orthoebolavirus bundibugyoense"  OR (BDBV AND (Ebola OR ebolavirus OR orthoebolavirus))) | 168 |
| N2-WO | Web of Science Core Collection | One Health, animal, reservoir, spillover and ecological surveillance | 12 Aug 2026 | ((TI=(Ebola OR ebolavirus* OR orthoebolavirus* OR "Bundibugyo virus" OR BDBV)  OR AB=("Bundibugyo virus" OR BDBV))  AND (TI=(reservoir* OR "natural host" OR "natural hosts" OR "animal host" OR "animal hosts"  OR bat OR bats OR wildlife OR zoono* OR spillover* OR "cross-species transmission"  OR "animal surveillance" OR "wildlife surveillance" OR "environmental surveillance" OR ecolog*)  OR AB=("natural host" OR "natural hosts" OR "animal host" OR "animal hosts"  OR "cross-species transmission" OR "animal surveillance" OR "wildlife surveillance"  OR "environmental surveillance")))  OR (TI=("One Health") AND TI=(definition OR "conceptual framework" OR "operational framework"))  OR (TI=(zoono*) AND TI=(spillover*) AND TI=(pathway* OR framework* OR driver*)) | 299 |
| N3-WO | Web of Science Core Collection | Preparedness, conflict-affected access, community engagement, IPC and cross-border response | 12 Aug 2026 | (TI=(Ebola OR ebolavirus* OR orthoebolavirus* OR "Bundibugyo virus" OR BDBV)  OR AB=("Bundibugyo virus" OR BDBV))  AND (TI=("outbreak preparedness" OR "outbreak response" OR "humanitarian emergency" OR "armed conflict"  OR "community engagement" OR mistrust OR "risk communication" OR "infection prevention and control"  OR "cross-border" OR "introduction risk" OR "health equity")  OR AB=("outbreak preparedness" OR "outbreak response" OR "humanitarian emergency" OR "armed conflict"  OR "community engagement" OR mistrust OR "risk communication" OR "infection prevention and control"  OR "cross-border" OR "introduction risk" OR "health equity")) | 338 |

*Note:* *No additional database limits were applied beyond the field specifications shown in the search strings. Searches were not limited by publication date, document type, language, full-text availability or subject area. All retrieved records were exported for deduplication and screening.*

**Table S4 documents the predefined selection criteria and the sources included outside the database-screening pathway.**

Table S4A. Eligibility and evidence-selection criteria

| **Assessment dimension** | **Criteria for retention** | **Reasons for non-advancement or exclusion** |
| --- | --- | --- |
| Temporal relevance | Evidence published from 2021 to the search date was prioritised for the current outbreak and contemporary preparedness. Evidence from 2014-2020 was retained when it provided transferable lessons from previous Ebola outbreaks or affected settings. Earlier publications were retained only when they provided non-substitutable foundational evidence on BDBV discovery, historical outbreaks, virology, diagnostics, medical countermeasures, reservoir ecology or a core conceptual framework. | Outside the temporal scope without current, transferable or foundational value. |
| Source type and informativeness | Eligible sources included original epidemiological or field studies; diagnostic, experimental, animal or preclinical studies; modelling analyses; evidence syntheses; implementation, policy or governance studies; and substantive letters, correspondence or short reports that contributed unique evidence. Official guidance and institutional reports were recorded separately from the database pathway. | News reports, general media or blogs without verifiable primary information; non-substantive commentary; conference records with insufficient information; and withdrawn, retracted or superseded versions. |
| Thematic and evidentiary relevance | Sources had to support a defined claim concerning BVD/BDBV, transferable orthoebolavirus evidence, One Health risk recognition, diagnostics, medical countermeasures, IPC and safe care, community engagement, conflict or access, cross-border continuity, or operational preparedness. | Merely mentioning Ebola, One Health or preparedness without contributing usable evidence to a defined review question. |
| Evidentiary directness and transferability | Direct BDBV evidence was prioritised. Evidence from EBOV, other orthoebolaviruses or other settings was retained only when the transferable mechanism and its limits could be stated explicitly. | Evidence concerning another pathogen, intervention or setting without a reasonable and clearly defined route of transfer to the BDBV preparedness question. |
| Verifiability, currency and non-redundancy | Bibliographic identity and relevant content had to be verifiable. Full text was examined for sources supporting substantive claims. When sources served the same function, preference was given to more direct, current, complete and non-redundant evidence. Non-English sources were retained only when relevant content could be reliably verified. | Relevant content could not be reliably verified; the source was retracted, withdrawn or replaced by a valid later version; or its contribution was fully duplicated by a stronger source. |

*Note: Publication type alone did not determine inclusion. Sources were retained for the narrative synthesis according to their relevance to the review questions, evidentiary directness and transferability, recency, verifiability and non-redundancy. Given the heterogeneous source types and the narrative purpose of the review, no formal risk-of-bias scoring tool was applied.*

Table S4B. Additional sources included outside the database-screening pathway

| **Revised-manuscript ref.** | **Source/title** | **Source category** | **Identification route** | **Main evidence function in the synthesis** |
| --- | --- | --- | --- | --- |
| 20 | Coarsey CT, et al. Strategies in Ebola virus disease (EVD) diagnostics at the point of care | Peer-reviewed review article | Targeted topic/DOI search | Transferable evidence on point-of-care diagnostic strategies and the placement of near-patient testing within the confirmation pathway. |
| 26 | Henao-Restrepo AM, et al. Efficacy and effectiveness of an rVSV-vectored vaccine in preventing Ebola virus disease: final results from the Guinea ring vaccination, open-label, cluster-randomised trial (Ebola Ça Suffit!) | Peer-reviewed clinical trial | Backward citation tracing | Foundational EBOV ring-vaccination efficacy evidence used to distinguish transferable vaccine evidence from direct BDBV efficacy evidence. |
| 43 | Wildlife health surveillance: gaps, needs and opportunities | Peer-reviewed review/policy article | Targeted topic/DOI search | Transferable implementation evidence on wildlife-surveillance capacity, data collection and cross-sector review. |
| 53 | MacNeil A, Reed Z, Rollin PE. Serologic cross-reactivity of human IgM and IgG antibodies to five species of Ebola virus | Peer-reviewed original study | Targeted topic/DOI search | Evidence on cross-reactive ebolavirus serology, used to constrain species-specific interpretation of field antibody signals. |
| 59 | Abdool Karim S, Faure M. The Bundibugyo Ebolavirus Outbreak: the need for a solidaristic response under the revised International Health Regulations | Peer-reviewed policy/commentary article | Targeted title search | Policy analysis supporting solidarity, equity and participation of affected regions in outbreak and research governance. |
| 60 | Sharan M, et al. Surveillance and response strategies for zoonotic diseases: a comprehensive review | Peer-reviewed review article | Targeted topic/DOI search | Transferable One Health surveillance and response principles, including shared governance and cross-sector coordination. |
| 64 | Kennedy SB, et al. Implementation of an Ebola virus disease vaccine clinical trial during the Ebola epidemic in Liberia: Design, procedures, and challenges | Peer-reviewed implementation study | Backward citation tracing | Transferable evidence for advance trial preparation, including protocols, trained sites and operational arrangements during an outbreak. |
| 1 | WHO. Ebola disease caused by Bundibugyo virus, Democratic Republic of the Congo & Uganda | WHO Disease Outbreak News | Official organisational source search | Initial outbreak scope, cross-border occurrence and current-event framing. |
| 6 | WHO. Experts convened by WHO advise on candidate treatments and vaccines for Ebola disease caused by Bundibugyo virus | WHO news/technical update | Official organisational source search | BDBV candidate treatment and vaccine prioritisation and MCM-readiness status. |
| 9 | WHO. Epidemic of Ebola disease caused by Bundibugyo virus in the Democratic Republic of the Congo and Uganda determined a public health emergency of international concern | WHO official announcement | Official organisational source search | Date and basis of the PHEIC determination used in the early-response timeline. |
| 10 | WHO. First meeting of the IHR Emergency Committee regarding the epidemic of Ebola Bundibugyo virus disease in the Democratic Republic of the Congo and Uganda 2026: temporary recommendations | WHO IHR Emergency Committee statement | Official organisational source search | Emergency Committee timing, temporary recommendations, notification and preparedness actions. |
| 11 | WHO/Africa CDC. Africa CDC and WHO launch joint continental Ebola response plan | Joint institutional announcement | Official organisational source search | Continental coordination and response-plan milestone. |
| 13 | WHO Regional Office for Africa. Ebola Bundibugyo virus disease outbreak, Democratic Republic of the Congo \| Uganda: Weekly External Situation Report 15 | Official situation report | Official organisational source search | Evidence-cut-off case totals, deaths, affected areas and geographic distribution. |
| 15 | WHO Regional Office for Africa. Uganda Hosts Regional Meeting to Boost Ebola Preparedness Amid Ongoing Outbreak | Official regional update | Official organisational source search | Regional and cross-border preparedness coordination milestone. |
| 18 | ECDC. Laboratory guidance and resources | ECDC technical webpage | Official organisational source search | Laboratory preparedness and testing-resource context for specimen confirmation. |
| 21 | WHO. Diagnostic testing for Ebola disease and Marburg virus disease: interim guidance, 9 July 2026 | WHO interim guidance | Official organisational source search | Standardised specimen collection, diagnostic analysis and result reporting. |
| 22 | WHO Prequalification. WHO lists the first BDBV test under its EUL procedure | WHO regulatory/prequalification update | Official organisational source search | First BDBV diagnostic Emergency Use Listing and regulatory-access milestone. |
| 35 | CEPI. CEPI fast-tracks three Bundibugyo ebolavirus vaccine candidates | CEPI programme announcement | Official organisational source search | BDBV vaccine-candidate prioritisation and development status. |
| 36 | WHO. Patient enrolment begins in a scientific trial to identify the first effective treatments for Bundibugyo virus disease | WHO trial update | Official organisational source search | Start of PARTNERS enrolment and activation of BDBV therapeutic evaluation. |
| 37 | WHO. WHO guidelines for the clinical management of filovirus disease | WHO clinical guideline | Official organisational source search | Supportive care, suspected-case management and IPC principles. |
| 47 | WHO. Risk communication and community engagement readiness and response toolkit: Ebola disease | WHO operational toolkit | Official organisational source search | Risk communication, community engagement and feedback functions. |
| 51 | ECDC. Preparedness and response for imported cases of Ebola disease into an EU/EEA country: operational checklists to support national preparedness planning | ECDC operational guidance | Official organisational source search | Transferable institutional interfaces for imported-case and cross-border preparedness. |
| 54 | ECDC. Rapid ECDC advice on infection prevention and control measures for Ebola disease in EU/EEA healthcare settings | ECDC technical advice | Official organisational source search | IPC and safe-management measures for suspected or confirmed cases. |
| 63 | International Pandemic Preparedness Secretariat. Fifth Implementation Report | Institutional preparedness report | Official organisational source search | Advance research and MCM preparedness, including pre-arranged protocols and response-ready evaluation capacity. |

*Note: Seven additional peer-reviewed reports and 17 official or institutional web sources were included. Together with 40 database-screened reports, they yielded 64 unique sources in the narrative synthesis. Official sources were used for current outbreak data, institutional milestones, operational guidance, and regulatory or product-development status; they were not counted as database records.*

**Abbreviations**

| **Abbreviation** | **Full term** |
| --- | --- |
| BDBV | Bundibugyo virus |
| BVD | Bundibugyo virus disease |
| CEPI | Coalition for Epidemic Preparedness Innovations |
| DOI | digital object identifier |
| DRC | Democratic Republic of the Congo |
| EBOV | Ebola virus |
| EUL | Emergency Use Listing |
| EVD | Ebola virus disease |
| IHR | International Health Regulations |
| IPC | infection prevention and control |
| MCM | medical countermeasure |
| NAAT | nucleic acid amplification test |
| PALM | Pamoja Tulinde Maisha |
| PARTNERS | Platform Adaptive Randomised Trial for New and Repurposed Filovirus Treatments |
| PCR | polymerase chain reaction |
| PHEIC | public health emergency of international concern |
| PMID | PubMed identifier |
| POCT | point-of-care testing |
| RCCE | risk communication and community engagement |
| RNA | ribonucleic acid |
| RT-qPCR | reverse-transcription quantitative polymerase chain reaction |
| WHO | World Health Organization |
